# Elevated serum adipocyte fatty acid-binding protein concentrations are independently associated with renal dysfunction in patients with stable angina pectoris

**DOI:** 10.1186/1475-2840-11-26

**Published:** 2012-03-21

**Authors:** Mutsumi Iwamoto, Toru Miyoshi, Masayuki Doi, Ko Takeda, Masahito Kajiya, Kazumasa Nosaka, Rie Nakayama, Satoshi Hirohata, Shinichi Usui, Shozo Kusachi, Kosuke Sakane, Kazuhfumi Nakamura, Hiroshi Ito

**Affiliations:** 1 Department of Cardiology, Kagawa Prefectural Central Hospital, Kagawa, Japan; 2 Department of Cardiovascular Therapeutics, Okayama University Graduate School of Medicine, Dentistry and Pharmaceutical Sciences, Okayama, Japan; 3 Department of Cardiovascular Medicine, Okayama University Graduate School of Medicine, Dentistry and Pharmaceutical Sciences, Okayama, Japan; 4 Department of Molecular Biology and Biochemistry, Okayama University Graduate School of Medicine, Dentistry and Pharmaceutical Sciences, Okayama, Japan; 5 Department of Medical Technology, Okayama University Graduate School of Health Sciences, Okayama, Japan

**Keywords:** Adipocyte, Fatty acid-binding protein, Renal dysfunction, Coronary artery disease

## Abstract

**Background:**

Chronic kidney disease (CKD) is associated with cardiovascular events. Adipocyte fatty acid-binding protein (A-FABP) plays an important role in atherosclerosis. We investigated whether plasma A-FABP is involved in renal function in patients with stable angina pectoris.

**Methods:**

A total of 221 patients with significant coronary artery stenosis were enrolled after coronary angiography. CKD was defined as an estimated glomerular filtration rate (eGFR) < 60 ml/min/1.73 m^2^. The severity of coronary stenosis was assessed using a modified Gensini score and coronary angiography. Serum A-FABP levels were determined by enzyme-linked immunosorbent assay.

**Results:**

Serum A-FABP levels were significantly correlated with both eGFR (r = -0.41, p < 0.01) and the severity of coronary artery stenosis (r = 0.16, p = 0.02), and these relationships remained significant after adjusting for confounding factors. The prevalence of CKD and multi-vessel disease was significantly higher among patients with serum A-FABP levels above the median value of 20.3 ng/ml than among patients with serum A-FABP levels below the median value (57% vs. 27%, p < 0.01 and 64% vs. 48%, p = 0.02, respectively). Multivariate analysis revealed that the presence of three-vessel disease in comparison with single-vessel disease was independently associated with the higher A-FABP (per doubling) (odds ratio; 2.26, 95% confidential interval; 1.28-3.98, p < 0.01) and tended to be associated with the lower eGFR (p = 0.06).

**Conclusion:**

Serum A-FABP may have a significant role in the interplay between renal dysfunction and coronary atherosclerosis.

## Background

Obesity and obesity-associated disorders, including insulin resistance, type 2 diabetes, dyslipidemia, and hypertension, are rapidly increasing in developed countries. In association with weight gain, the hyperplasia and hypertrophy of adipocytes influence the secretion pattern of adipocyte-derived proteins, adipokines, by adipose tissue. Recent evidence shows that adipokines contribute to the increased metabolic and cardiovascular risk among obese patients [1]. Among those adipokines, adipocyte fatty acid-binding protein (A-FABP), also known as aP2 or FABP4, is small intracellular lipid-binding protein which is expressed abundantly in adipocytes and activated macrophages [2]. Now, there are nine types of FABPs, showing tissue-specific expression patterns, and several members of the FABP family have been shown to have important roles in regulating metabolism and have links to the development of insulin resistance and the metabolic syndrome [3]. A-FABP was reported to play an essential regulatory role in energy metabolism and inflammation [4], and found not only in tissue, but also in blood stream [5].

The pathophysiological role of A-FABP has been investigated in murine experimental models and clinical studies. In mice, A-FABP deficiency ameliorates the development of insulin resistance in diet-induced obesity [2], type 2 diabetes [6], and atherosclerosis in models of hypercholesterolemia [7]. Clinically, A-FABP is detected in human serum [5]. Higher serum A-FABP levels are used to predict and diagnose obesity-related metabolic syndrome and type 2 diabetes [8,9]. Previous studies also showed that serum A-FABP levels are associated with carotid intima-media thickness [10], coronary artery disease [11], the number of stenotic coronary arteries [12], and coronary plaque volume, as determined by intravascular ultrasound [13]. Furthermore, the involvement of A-FABP in atherosclerosis is supported by a genetic study in humans. Carriers of the T87C polymorphism have lower serum triglyceride levels, demonstrating a reduced cardiovascular risk [14]. These findings demonstrate that A-FABP may play a critical role in the development of metabolic syndrome, type 2 diabetes, and cardiovascular disease.

Although the association between A-FABP and several metabolic parameters has been studied in detail, little is known about the relationship between this adipokine and renal function. One study showed that serum A-FABP concentrations in patients with chronic hemodialysis are higher than those in control patients without hemodialysis [15], although serum A-FABP levels in patients with a mild to moderate decrease in glomerular filtration rate (GFR) remain untested. Furthermore, the association between serum A-FABP, eGFR, and severity of coronary artery disease has not been evaluated. Therefore, we determined serum A-FABP levels in 221 patients with stable angina pectoris and assessed the correlation between serum A-FABP levels and biochemical measures of renal function, as well as the severity of coronary artery disease.

## Methods

### Study group

This study included 221 patients with stable angina pectoris who underwent coronary angiography between April 2008 and September 2009 at Kagawa Prefectural Central Hospital, Japan. Patients who had 75% or greater organic stenosis of at least one major coronary artery or who had previously undergone percutaneous transluminal coronary angioplasty were included. Patients with chronic hemodialysis, acute coronary syndrome, recent (within 4 weeks) myocardial infarction, or malignancies were excluded. The study protocol complied with the Declaration of Helsinki and was approved by the Ethics Committees of the institute. Written informed consent was obtained from all patients before study enrollment.

### Clinical and biochemical assessments

Blood samples were taken after overnight fasting. The serum was separated and stored at -80°C, and serum levels of A-FABP (Biovendor Laboratory Medicine, Modrice, Czech Republic) and high-sensitivity C-reactive protein (hs-CRP; R&D Systems, Minneapolis, MN, USA) were measured by enzyme-linked immunosorbent assay [13]. The performance characteristics of these assays were < 7% and < 8% intra-assay coefficient of variation (CV), and < 5% and < 7% inter-assay CV for A-FABP and hs-CRP, respectively.

Risk factors were defined as follows. Diabetes was confirmed using the criteria of the American Diabetes Association [16] or by a history of treatment for diabetes mellitus. Dyslipidemia was defined as one or more of the following criteria: (1) serum triglyceride ≥ 150 mg/dl; (2) high-density lipoprotein (HDL)-cholesterol < 40 mg/dl; (3) low-density lipoprotein (LDL)-cholesterol ≥ 140 mg/dl; and (4) current use of lipid-lowering medication. Hypertension was defined as a sitting blood pressure ≥ 140/90 mmHg or current use of antihypertensive medication. Smoking status was determined and classified as current smoker or not. The estimated GFR (eGFR) was calculated using the equation put forth by the Modification of Diet in Renal Disease (MDRD) Study Group [17], with coefficients modified for Japanese patients [18]: eGFR (ml/min/1.73 m^2^) = 194 × (serum creatinine)^-1.094 ^× (age)^-0.287 ^(x0.739 if female). The distribution of the eGFR was divided into three categories: less than 60 (moderately decreased eGFR, n = 93), 60-89 (mildly decreased eGFR, n = 106) and at least 90 ml/min/1.73 m^2 ^(normal eGFR, n = 22). Patients with end stage renal disease were not included. Chronic kidney disease (CKD) was defined as eGFR < 60 ml/min/1.73 m^2^.

### Coronary angiography

Coronary angiography was performed according to standard methods. After intracoronary injection of isosorbide dinitrate, angiograms were obtained in two or more views. The coronary angiogram was scored by two independent investigators. The stenosis score is a modified Gensini score [19]. Briefly, the most severe stenosis in each of eight segments was graded according to severity, from 1 to 4. The scores in each of the eight segments were added to provide a total stenosis score out of a maximum of 32.

### Statistical analysis

Continuous variables are presented as mean ± standard deviation (SD) or median (interquartile range [(IQR]) values, and differences between groups were analyzed using an unpaired Student's *t *test. Data that were not normally distributed, as determined using the Kolmogorov-Smirnov test, were logarithmically transformed before linear regression analysis. Categorical variables are presented as frequency counts and corresponding percentages, and intergroup comparisons were analyzed using the chi-square test. Associations between serum A-FABP and other parameters were first analyzed by simple linear regression analysis and then by multivariate logistic regression analysis. To assess the association between serum A-FABP level and the presence of CKD or three-vessel coronary artery disease, logistic regression analyses were performed. In those analyses, factors that were associated with the dependent variable at p < 0.05 in the univariate analysis were entered into the multivariate model. In multivariate model, diabetes was selected as a covariate because fasting glucose levels, hemoglobinA1c, the homeostasis model assessment ratio are confounding factors of diabetes. Statistical significance was defined as p < 0.05. Statistical analyses were performed using SPSS 16.0 for Windows (SPSS Inc., Chicago, IL, USA).

## Results

### Patient characteristics

**Figure 1:**
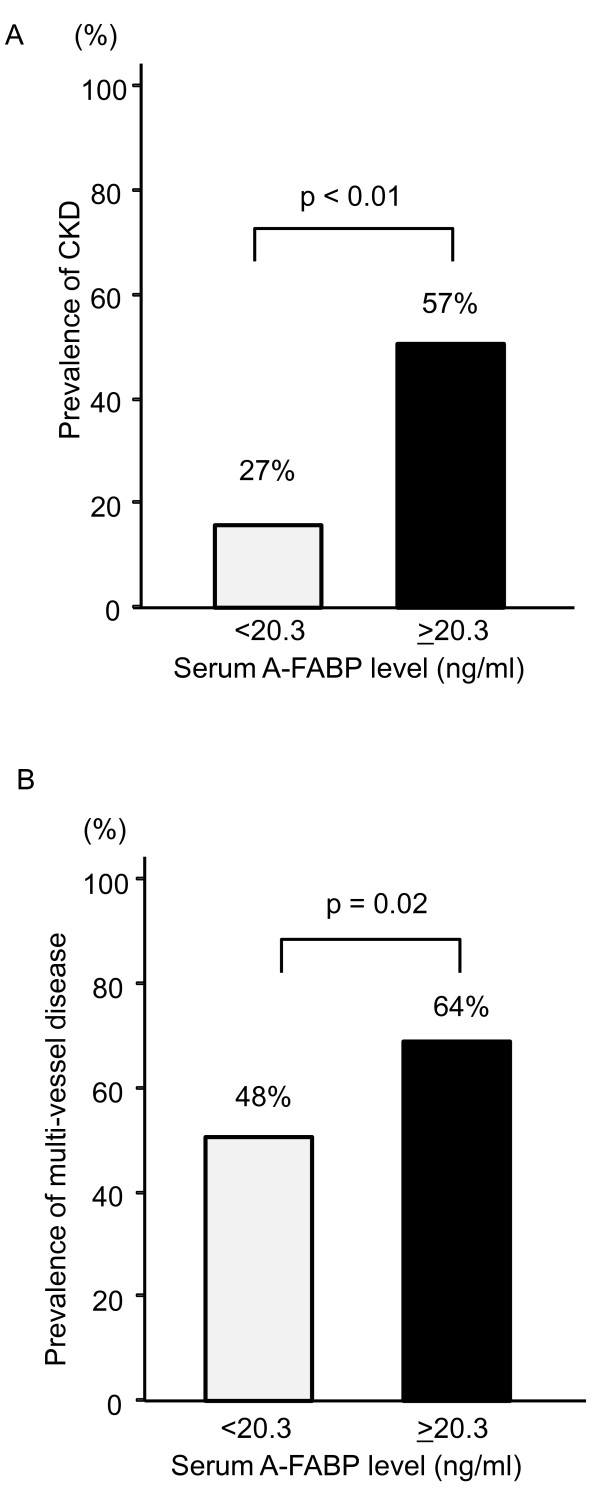
**Prevalence of CKD (A) and multi-vessel disease (B) among patients grouped according to the median value of serum A-FABP (20.3 ng/ml)**.

The clinical characteristics of the study population are shown in Table 1. Patients with eGFR levels < 60, 60-89, or > 90 ml/min/1.73 m^2 ^differed in age, the presence of hypertension, smoking status, serum triglycerides levels, uric acid levels, fasting glucose levels, hemoglobinA1c, the homeostasis model assessment ratio (HOMA-R), and serum A-FABP levels but not in the number of diseased vessels or the stenosis score. The eGFR value was significantly lower among patients with hypertension than among patients without hypertension (mean ± SD, 66.3 ± 18.1 ml/min/1.73 m^2 ^vs. 73.1 ± 18.6 ml/min/1.73 m^2^, p < 0.01 by Student's *t *test). The eGFR value was significantly higher among smokers than among non-smokers (76.1 ± 17.3 ml/min/1.73 m^2 ^vs. 63.9 ± 18.5 ml/min/1.73 m^2^, p < 0.01). The eGFR value did not vary by the presence or absence of diabetes mellitus or dyslipidemia, gender, or the use of specific medications (data not shown). The stenosis score was significantly higher among patients with diabetes mellitus than among patients without diabetes mellitus (1.9 ± 0.8 vs. 1.6 ± 0.7, p < 0.01). The stenosis score did not vary by the presence or absence of hypertension or dyslipidemia, smoking status, gender, or the use of specific medications (data not shown). The prevalence of CKD and multi-vessel disease based on the median value of serum A-FABP (20.7 ng/ml) were shown in Figures 1A and 1B. The prevalence of CKD and multi-coronary vessel disease was significantly higher among patients with serum A-FABP levels over the median value than among patients with serum A-FABP levels less than the median value (57% vs. 27%, p < 0.01 and 64% vs. 48%, p = 0.02, respectively).

**Table 1:** Patient characteristics in this study

		eGFR (ml/min/1.73 m^2^)			
			
	ALL	< 60	60-89	≥ 90	
	(n = 221)	(n = 93)	(n = 106)	(n = 22)	p
Age (years)	71 ± 10	76 ± 8	68 ± 10	62 ± 11	< 0.01
Male, n (%)	185(84)	74(80)	92(87)	19(86)	0.36
Body mass index (kg/m^2^)	24.7 ± 3.6	24.5 ± 3.7	25.0 ± 3.4	24.0 ± 4.5	0.29
Hypertension, n (%)	157(71)	77(83)	77(73)	12(55)	0.02
Dyslipidemia, n (%)	183(83)	75(81)	89(84)	19(86)	0.72
Diabetes Mellitus, n (%)	96(43)	31(33)	53(50)	12(54)	0.03
Smoking (Yes)	28(13)	5(5)	18(16)	5(23)	0.02
LDL-Cholesterol (mg/dl)	102 ± 28	100 ± 28	103 ± 29	10.4 ± 26	0.77
HDL-Cholesterol (mg/dl)	43 ± 12	42 ± 11	44 ± 12	44 ± 11	0.59
Triglycerides (mg/dl)	163(79)	155(107)	169(61)	167(68)	< 0.01
Uric acid (mg/dl)	5.8 ± 1.6	6.4 ± 1.6	5.5 ± 1.4	4.9 ± 1.6	< 0.01
Fasting blood glucose (mg/dl)	100(25)	96(18)	102(34)	104(28)	< 0.01
HOMA-R	1.7(1.5)	1.7(1.3)	1.8(1.7)	1.3(1.1)	< 0.01
HemoglobinA1c (%)	5.6(1.2)	5.4(0.8)	5.7(1.2)	5.9(1.0)	0.04
hs-CRP(mg/l)	0.97(2.35)	1.25(3.27)	0.82(1.79)	1.59(2.72)	0.10
Serum A-FABP (ng/ml)	20.3(13.6)	26.9(19.5)	19.6(11.1)	16.1(4.7)	< 0.01
Number of diseased vessels	1.8 ± 0.8	1.9 ± 0.8	1.7 ± 0.8	1.5 ± 0.7	0.12
Stenosis score	9.9 ± 4.9	9.3 ± 4.5	9.5 ± 5.8	10.2 ± 5.1	0.71
*Medications*	28(13)	5(5)	18(16)	5(23)	0.02
ACEI/ARB, n (%)	119(54)	39(43)	66(62)	14(64)	0.18
CCBs, n (%)	120(54)	48(52)	57(38)	15(68)	0.39
β-blockers, n (%)	73(33)	27(29)	40(38)	6(27)	0.36
Statins, n (%)	126(57)	49(52)	63(59)	14(63)	0.50

Data are presented as the mean ± SD, median (IQR), or frequency counts (percentages), as appropriate. LDL-C, low-density lipoprotein cholesterol; HDL-C, high-density lipoprotein cholesterol; FBS, fasting blood glucose; HOMA-R, homeostasis model assessment ratio; hs-CRP, high-sensitivity C-reactive protein; eGFR, estimated glomerular filtration rate; A-FABP, adipocyte fatty acid-binding protein: ACEI, angiotensin-converting enzyme inhibitor; ARB, angiotensin II receptor blocker; CCBs, calcium channel blockers

### Serum A-FABP levels and other biochemical parameters

**Figure 2:**
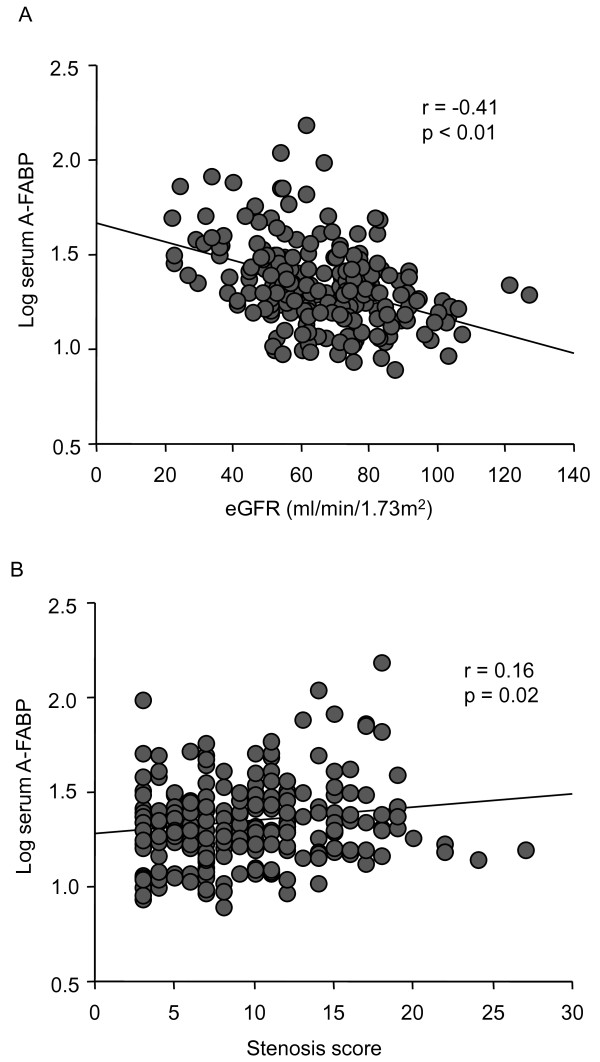
**Correlation between serum A-FABP levels and eGFR (A) and coronary stenosis score (B) (n = 221)**.

Serum A-FABP levels were significantly higher among females than among males (median (IQR), 30.9 (26.7) ng/ml vs. 19.79(11.5) ng/ml, p < 0.01). Serum A-FABP levels were also significantly higher in patients with hypertension than those without hypertension (21.4 (14.9) ng/ml vs. 18.5(13.3) ng/ml, p = 0.02). Serum A-FABP levels did not vary by the presence or absence of diabetes mellitus, dyslipidemia, smoking status, or the use of specific medications (data not shown). As shown in Table 2 and Figure 2, serum A-FABP levels correlated significantly with gender, eGFR levels (Figure 2A), body mass index, hs-CRP levels, and stenosis scores (Figure 2B). Multiple linear regression analysis revealed that the serum A-FABP level was independently associated with the eGFR value and the stenosis score along with gender or body mass index. Next, the associations between CKD and other biochemical parameters were assessed (Table 3). Multiple logistic regression analysis revealed that the serum-A-FABP level (per doubling) was independently associated with CKD, with an odds ratio of 3.7 (95% confidential interval; 2.14-6.461, p < 0.01). Finally, the associations of the severity coronary artery disease with the levels of eGFR and serum A-FABP were analyzed by logistic regression analysis (Table 4). Multivariate analysis revealed that the presence of three-vessel disease in comparison with single-vessel disease was independently associated with the higher A-FABP level(per doubling) (odds ratio; 2.26, 95% confidential interval; 1.28-3.98, p < 0.01) and tended to be involved in the lower eGFR value (per ml/min/1.73 m^2^)(odds ratio; 0.98, 95% confidential interval; 0.96-1.00, p < 0.06).

**Table 2:** Relationship between serum A-FABP and other parameters

	Univariate		Multivariate	
	
	r	p	β	p
Age	0.09	0.14		
Gender (male = 1)	-0.33	< 0.01	-0.31	< 0.01
Body mass index	0.35	< 0.01	0.35	< 0.01
Uric acid	0.12	0.08		
LDL-cholesterol	0.06	0.37		
HDL-cholesterol	-0.12	0.09		
Triglycerides*	-0.05	0.50		
HbA1c*	0.06	0.46		
HOMA-R*	0.11	0.10		
hs-CRP*	0.15	0.03	0.05	0.32
eGFR	-0.41	< 0.01	-0.40	< 0.01
Stenosis score	0.16	0.02	0.15	< 0.01

Values indicated with * were included in the model after log-transformation. In the model, R^2 ^= 0.42

**Table 3:** Relationship between CKD and other parameters

	Crude		Adjusted	
		
Factors	OR (95%CI)	p	OR (95%CI)	p
Age (per year)	1.11(1.08-1.16)	< 0.01	1.11(1.07-1.17)	< 0.01
Male	0.59(0.29-1.22)	0.16		
Smoking (yes)	0.28(0.10-0.76)	0.01	0.44(0.13-1.49)	0.18
Hypertension (yes)	2.19(1.13-4.29)	0.03	1.25(0.54-2.914)	0.56
Diabetes (yes)	0.53(0.31-0.93)	0.03	0.62(0.29-1.33)	0.22
Fasting blood glucose (per doubling)	0.16 (0.05-0.46)	< 0.01		
HOMA-R* (per doubling)	0.99(0.78-1.27)	0.97		
HbA1c (per doubling)	0.192(0.05-0.74)	0.02		
Dyslipidemia	0.77(0.38-1.56)	0.47		
HDL (per mg/dl)	0.99(0.97-1.01)	0.31		
LDL (per mg/dl)	0.99(0.98-1.00)	0.49		
Triglycerides (per doubling)	0.44(0.27-0..71)	< 0.01	0.49(0.26-0.93)	0.03
Uric acid (per mg/dl)	1.59(1.29-1.94)	< 0.01	1.70 (1.02-2.22)	< 0.01
A-FABP (per doubling)	3.03(1.94-4.72)	< 0.01	3.14(1.89-5.31)	0.01

In the model, R^2 ^= 0.34

**Table 4:** Relationship between severe coronary artery disease and other parameters

	Crude		Adjusted	
		
Factors	OR (95%CI)	p	OR (95%CI)	p
Age (per year)	1.01(0.98-1.04)	0.55		
Male	1.39(0.51-3.79)	0.51		
Smoking (yes)	1.16(0.39-3.40)	0.79		
Hypertension (yes)	1.63(0.69-3.84)	0.26		
Diabetes (yes)	2.63(1.29-5.36)	< 0.01	3.20(1.43-7.17)	< 0.01
FBS (per doubling)	3.6(1.26-10.53)	0.02		
HOMA-R (per doubling)	1.31(0.95-1.80)	0.10		
HbA1c (per doubling)	5.95(1.21-29.14)	0.03		
Dyslipidemia	1.72(0.64-4.66)	0.28		
Triglycerides (per doubling)	1.11(0.63-1.95)	0.72		
HDL (per mg/dl)	0.97(0.94-1.00)	0.05		
LDL (per mg/dl)	0.99(0.98-1.00)	0.16		
Uric acid (per mg/dl)	1.02(0.82-1.27)	0.87		
eGFR (per ml/min/1.73m2)	0.97(0.95-0.99)	< 0.01	0.98(0.95-1.00)	0.06
A-FABP (per doubling)	2.97(1.77-4.98)	< 0.01	2.26(1.28-3.98)	0.01

In the model, R^2 ^= 0.16

## Discussion

We demonstrated that the serum A-FABP level was independently correlated with the eGFR value in patients with stable angina pectoris without hemodialysis. Serum A-FABP may be a novel marker of renal function as well as the severity of coronary artery disease in patients with a mild to moderate decrease in eGFR. Our findings suggest that circulating A-FABP may have an important role in the interplay between renal dysfunction and the development of coronary atherosclerosis.

The mechanism underlying the relationship between A-FABP and eGFR has not been fully clarified. Sommer et al. reported that serum A-FABP levels are more than 10-fold higher among patients with chronic hemodialysis than among controls [15]. In addition to A-FABP, circulating levels of adiponectin, leptin, and retinol-binding protein 4 have been reported to be higher among patients with chronic hemodialysis than among controls [20-22]. These results suggest that renal elimination plays an important role in determining the serum concentration of various adipocyte-derived proteins, including adiponectin, leptin, retinol-binding protein 4, and A-FABP, although the causal relationship between the elevated circulating A-FABP and renal dysfunction remains unclear. More mechanistic studies involving animal experiments are necessary to prove the concept that A-FABP is not only secreted from adipose tissue but also is cleared by the kidneys. Furthermore, markers of renal function should be included in future studies as potential confounders when examining the physiology and regulation of A-FABP in humans.

The finding that serum A-FABP was independently associated with the severity of coronary atherosclerosis is in agreement with our previous findings [11]. In human, recent study showed that circulating A-FABP levels were shown to be associated with vascular inflammation, as measured using (18)F-fluorodeoxyglucose positron emission tomography [23]. Peeters et al. reported that serum A-FABP levels and A-FABP concentrations in human carotid tissue were associated with the vulnerability of carotid plaques [24]. On the other hand, experimental studies showed that A-FABP plays a critical role in the development of atherosclerosis by coordinating the cholesterol-trafficking and inflammatory activity of macrophages [25]. A-FABP deficiency reduces foam cell formation in response to oxidized LDL and increases the cholesterol efflux pathway [25]. A-FABP-deficient mice also show a significant decrease in vascular atherosclerosis in the absence of differences in serum lipid levels or insulin sensitivity in a model of hyperlipidemia, and this effect is due OR has been attributed to the effects of A-FABP on macrophages [7]. In addition, A-FABP can activate several key inflammatory pathways. In A-FABP-deficient macrophages, the activity of the peroxisome proliferator-activated receptor γ and the liver X receptor α is enhanced, leading to suppressed transcription of several inflammatory genes [26,27]. In addition, the NF-κB pathway is impaired, resulting in suppression of inflammatory function [25].

The physiological significance of increased serum A-FABP in renal failure remains to be elucidated. CKD is strongly associated with the development of atherosclerotic lesions and mortality from cardiovascular disease [28]. Because A-FABP has been reported to induce dyslipidemia and atherosclerosis in animal models [7], A-FABP may contribute to the significantly increased cardiovascular mortality among patients with CKD. Recently, Furuhashi et al. reported that the circulating A-FABP level is a predictor of cardiovascular events in end-stage renal disease [29]. Peeters et al. also reported that the serum A-FABP levels in human carotid athermanous plaques were associated with adverse cardiovascular events [30]. Regarding a circulating FABP, recent studies showed heart -FABP may represent a marker for early atherosclerosis [31]. Thus, the roles of FABPs as a predictor of cardiovascular events are promising. Taken together with our findings, the elevated serum A-FABP in patients with CKD may be involved in plaque vulnerability in atherosclerotic lesions and may predict a future cardiovascular event.

The role of circulating A-FABP as an atherogenic factor remains unknown. A recent study reported that A-FABP directly and acutely depresses the contraction of cardiomyocytes by decreasing intracellular Ca^2+ ^levels [32], suggesting that a direct bioactive role for A-FABP may exist in cells. It is well established that A-FABP is expressed by adipocytes, which may be major contributors to circulating A-FABP levels. Therefore, A-FABP secreted from adipose tissue may contribute to the development of atherosclerosis. Future studies should address whether circulating A-FABP induces atherosclerosis by activating macrophages and vascular cells.

## Limitations

This study has several limitations that should be considered when interpreting the results. First, the sample size was not large. Second, our study was cross-sectional, which does not allow us to determine if a causal relationship exists between A-FABP and renal dysfunction or between A-FABP and the development of coronary artery disease. Prospective population-based studies are needed to address whether serum A-FABP is a risk factor for CKD or coronary artery disease. Finally, we enrolled patients who were admitted to the hospital for coronary angiography in order to obtain more accurate data on coronary stenosis. Most of our patients had established risk factors for coronary artery disease, and so, the generalizability of our findings to other patient populations is unclear.

## Conclusions

We demonstrated that the serum A-FABP level was independently associated with CKD. Serum A-FABP may be a marker of renal dysfunction and may be associated with the severity of coronary artery disease in patients with a mild to moderate decrease in eGFR. Thus, circulating A-FABP may have an important role in the interplay between renal dysfunction and the development of coronary atherosclerosis. Further studies with larger cohorts derived from the general population are necessary to evaluate whether circulating A-FABP levels can be used to predict the risk of renal dysfunction and the development of coronary artery disease.

## Abbreviations

A-FABP: Adipocyte fatty acid-binding protein; ACEI: Angiotensin-converting enzyme inhibitor; ARB: Angiotensin II receptor blocker; CCBs: Calcium channel blockers.; CKD: Chronic kidney disease; CV: intra-assay coefficient of variation; eGFR: estimated glomerular filtration rate; FBS: fasting blood glucose; HDL-C: high-density lipoprotein cholesterol; HOMA-R: homeostasis model assessment ratio; hs-CRP: high-sensitivity C-reactive protein; IQR: interquartile range; LDL-C: low-density lipoprotein cholesterol.

## Competing interests

The authors declare that they have no competing interests.

## Authors' contributions

MI, TM, MD, KT, MK, KN, SK and RN conceived the study, participated in study design and coordination, and assisted with the preparation of this manuscript. SU conducted the immunoassays. SH, SK, KN, and HI assisted with the preparation or critical review of this manuscript. All authors read and approved the submitted manuscript.
